# Methodology of clinical trials on sodium-glucose cotransporter 2 inhibitors registered on ClinicalTrials.gov: a cross-sectional study

**DOI:** 10.1186/s12874-024-02292-5

**Published:** 2024-07-30

**Authors:** Fran Šaler, Marin Viđak, Livia Puljak

**Affiliations:** 1 Department of Cardiovascular Medicine, Clinical Hospital Dubrava, Zagreb, Croatia; 2 Center for Evidence-Based Medicine and Healthcare, Catholic University of Croatia, Zagreb, Croatia

**Keywords:** SGLT2 inhibitors, ClinicalTrials.gov, Clinical trials, Diabetes, Heart failure

## Abstract

**Background/Objective:**

The research on sodium-glucose cotransporter 2 (SGLT2) inhibitors has been increasing rapidly in the last decade, as well as indications for their use. This study aimed to analyze the methodological characteristics of clinical trials on SGLT2 inhibitors registered on ClinicalTrials.gov.

**Design:**

We conducted a cross-sectional study of trials on SGLT2 inhibitors registered on ClinicalTrials.gov up to November 11, 2022. We included clinical trials that tested SGLT2 inhibitors for any clinical condition, as a single or combined SGLT2 therapy, compared to any other medication or placebo and mapped their characteristics.

**Results:**

We identified 1102 eligible trials on 14 different SGLT2 inhibitors. The first trial registration was in 2005. There were 993 (90%) interventional and 109 (10%) observational trials. Most trials were in Phase 1 (29%), Phase 3 (23%), or Phase 4 (24%). Interventional trials were mostly randomized (85%); almost half of them did not use masking (44%). Trials on empagliflozin, dapagliflozin, and canagliflozin accounted for 75% of all trials. More than 60% of trials included patients with diabetes mellitus, 13% included only healthy subjects, and 12% included patients with heart diseases. Overall, these trials included more than 9.5 million participants (~ 312,000 of which in interventional studies). Almost 65% of all clinical trials were industry-funded. Most trials were completed (60%) and 35% of those reported results. For trials that are obligated to report results by the Food and Drugs Administration (FDA), 88% of them did so. Trials fully or partially funded by industry more frequently published results compared to non-industry funded trials (46.1% vs. 11.2%; *p* < 0.001).

**Conclusions:**

The number of registered trials on SGLT2 inhibitors is increasing progressively along with expanding indications for its use, shifting from diabetes mellitus to cardiovascular and renal diseases. Public reporting of trial results improved with time but remains suboptimal.

**Supplementary Information:**

The online version contains supplementary material available at 10.1186/s12874-024-02292-5.

## Background

Cardiovascular diseases and diabetes mellitus (DM) are leading global causes of mortality and morbidity with ever-increasing prevalence [1]. Identification of sodium-glucose cotransporter 2 (SGLT2) in renal tubules helped develop a new class of medicines that modify renal glucose metabolism – SGLT2 inhibitors [2]. Initially developed for glycemic regulation, SGLT2 inhibitors displayed cardiorenal protective effects through complex and multifactorial mechanisms, warranting further research [3].

In the last decade, new medicines targeting SGLT2 have gained approval, while many are still in development [4]. This group of pharmaceuticals made a breakthrough in the field of heart failure (HF) when clinical trials demonstrated that SGLT2 inhibitors independently reduce cardiovascular death and hospitalization for HF in diabetic and non-diabetic patients [5, 6].

The use of medications for conditions outside of their regulated indications, ‘off-label’, is common practice in medicine. Many specialties share a pool of medications for different indications, primarily immunology and oncology [7], and SGLT2 inhibitors are now expanding this collaboration between endocrinology and cardiology. Trials now explore a wide range of outcomes to maximize the potential use of medications, and some trials, like RECOVERY in the COVID-19 pandemic, test a variety of medications for a single condition [8, 9]. The expansion of indications promises improvements for many conditions, and makes the transparency of trials even more important than before.

Detailed and clear reporting of clinical trial methodology and results are vital in critical evaluation of evidence and its translation to clinical practice [10]. Prospective registration of clinical trials can help reduce questionable research practices and publication bias [11–13]. One of the largest publicly available clinical trial registries is ClinicalTrials.gov [14], which includes trial registrations from 50 states and 221 countries worldwide. Clinicaltrials.gov is a comprehensive database of privately and publicly funded clinical studies conducted around the world, maintained by the U.S. National Library of Medicine and is considered to be most explored and most informative clinical trial registry [15].

In 2007, The Food and Drugs Administration (FDA) implemented regulatory requirements for submitting registration and result information – Sect. 801 of the FDA Amendments Act (FDAAA 801) [16]. This act required that trials must be registered on ClinicalTrials.gov no later than 21 days after enrolment of the first participant and that results must be posted no later than 1 year after the study completion [16]. Trials on medication, devices, or biological agents, with at least one US site, of Phase 2 or later, or an FDA approved medications – are obligated to publish the results on ClinicalTrials.gov [16]. This was expanded in 2017 (42 CFR Part 11) with the obligation to provide supplementary documents for applicable trials with a primary completion date on or after January 17, 2017 [17].

Analysis of registered trials on newly developed medicines can provide insight into design, development, and potential pitfalls. Anticipatory thinking that takes into account potential risks and possible benefits is an ethical requirement in designing and conducting trials of new medications. To our knowledge, there are no published studies on methodology and design of registered clinical trials on SGLT2 inhibitors.

The aim of this study was to analyze the methodological characteristics of clinical trials on SGLT2 inhibitors registered on ClinicalTrials.gov.

## Methods

### Study design and setting

This was an observational, cross-sectional study of clinical trials on SGLT2 inhibitors registered at the ClinicalTrials.gov registry. We used the STROBE (Strengthening the reporting of observational studies in epidemiology) checklist for reporting our results [18]. Protocol for the study is available on the Open Science Framework at: https://osf.io/6vt3x/.

### Eligibility criteria

We included all trials on SGLT2 inhibitors available on ClinicalTrials.gov for any clinical condition, as a single or combined SGLT2 therapy, regardless of the study design. Fourteen SGLT2 inhibitors were included, namely bexagliflozin, canagliflozin, dapagliflozin, empagliflozin, enavogliflozin, ertugliflozin, henagliflozin, ipragliflozin, licogliflozin, luseogliflozin, remogliflozin, sergliflozin, sotagliflozin, tofogliflozin (Table 1) [4, 19–22].

**Table 1:** SGLT2 inhibitors included in the analysis, sorted chronologically based on the date of the first trial registered

SGLT2 inhibitor	Code	Date of the first trial
Dapagliflozin	BMS-512,148	13/09/2005
Remogliflozin	GSK189075	14/02/2006
Sergliflozin	GW869682	28/02/2006
Empagliflozin	BI 10,773	15/11/2007
Ipragliflozin	ASP1941	22/02/2008
Canagliflozin	JNJ-28,431,754, TA-7284	25/03/2008
Bexagliflozin	EGT1442, EGT-1474, THR-1442	02/03/2009
Sotagliflozin	LX4211	19/08/2009
Ertugliflozin	PF-04971729, MK-8835	02/10/2009
Licogliflozin	LIK066	01/08/2011
Tofogliflozin	CSG452	25/07/2014
Henagliflozin	SHR-3824	26/01/2015
Luseogliflozin	TS-071, 898537-18-3	16/07/2015
Enavogliflozin	DWP16001, DWP-16,001	07/12/2017

There were no time or geographical limitations. We excluded trials where SGLT2 inhibition was achieved using genetic modifiers of the SGLT2 receptor, trials on diagnostic and genetic tests, procedures, and dietary supplements.

### Search

We designed a search strategy by combining keywords pertaining to (1) pharmacological group, (2) generic names of SGLT2 inhibitors, and (3) code names of SGLT2 inhibitors used in early phases of medicine development. Generic names of SGLT2 inhibitors and code names were identified by screening relevant systematic reviews on Medline [4, 19–22] as well as the MeSH SGLT2 inhibitors category. Full text of the search strategy is available in Supplementary Table 1.

### Screening

The database search was performed on November 11, 2022. By using the website in-built option, search results were extracted, containing all available trial registry data, and all retrieved registry items were exported to Microsoft Excel. Deduplication was performed with the Excel function ‘’Remove duplicates’’, using the NCT number as a unique identifier. During coding and database analysis, two additional SGLT2 inhibitors were identified that were previously not included in our search protocol (enavogliflozin and henagliflozin). The search was repeated on March 19, 2023, to add these two medicines (including their medicinal codes) into the database of trials registered by November 11, 2022. Two authors (FŠ, MV) independently screened the search results. There were no disagreements between the authors during the screening, so there was no need for arbitration by the third author.

### Study outcomes

We analyzed the following primary outcome measures: Study type (Interventional; Observational; Expanded Access); Intervention (Types of SGLT2 inhibitors studied); Indication (Diabetes mellitus type 1, Diabetes mellitus type 2, Heart Failure, Atrial fibrillation, Cardiovascular Diseases, Chronic Kidney Disease, Healthy Subjects, Coronary Artery Disease, Myocardial Infarction, Obesity, Other); Comparators (SGLT2 inhibitor; Placebo; Gold standard therapy); Primary purpose (basic science, Diagnostic, Prevention, Screening, Supportive Care, Treatment); Enrollment data (Sample Size, Age, Gender); Blinding/masking [(None; Single (Participant; Investigator; Outcome assessor); Double (Participant-Investigator; Participant-Outcomes assessor; Participant-Care provider; Investigator-Outcome Assessor); Triple (Participant-Care Provider-Investigator; Participant-Care Provider-Outcomes assessor; Participant-Investigator-Outcomes assessor); Quadruple)]; Status (Not yet recruiting; Active, not yet recruiting; Active, not recruiting; Enrolling by invitation; Recruiting; Suspended; Terminated; Withdrawn; Completed; Unknown status); Trial phases (Not applicable, Early Phase 1, Phase 1, Phase 1/Phase 2, Phase 2, Phase 2/Phase 3, Phase 3, Phase 4); Posting of results (Has results, No results available).

We analyzed the following secondary outcome measures: Funding (sponsors/collaborators); Duration; Date first posted; Location.

### Hypotheses

We hypothesized that in clinical trials on SGLT2 inhibitors registered on ClinicalTrials.gov more than 50% of trials: will analyze medicine empagliflozin and dapagliflozin; will be interventional studies; will be trials in diabetes mellitus; will be sponsored by industry (commercial sponsors); will not have study results posted; and will have recruitment status “Completed”.

### Data extraction

The data charting form was modeled after the data items available at ClinicalTrials.gov for the registered trials. One author (FŠ) exported all available data columns from ClinicalTrials.gov. Some data were not available or were missing in the exported table. Thus, data items were checked and extracted manually by one author (FŠ), where needed. Reasons for termination, withdrawal, or suspension were extracted manually. Category “Conditions” was manually corrected as 82 trials had incorrect inputs. There were several incorrect extractions in the category “Interventions,” e.g., transposition of study medication and comparator, missing data, and incorrect data input. These categories required most of the manual corrections. Applicable clinical trials according to FDAAA 801 rule were identified, categorized, and analyzed separately. Risk of bias assessment was not done in this study.

### Statistical analysis

Statistical analysis was conducted using JASP (version 0.17.1). Descriptive statistics were used to present the collected data. Categorical variables were presented as frequencies and percentages; continuous variables were presented as median values and interquartile range. To test differences between groups for categorical data, the chi-squared test was used. The level of statistical significance was set for p-value < 0.05.

### Raw data

All raw data collected within this study, as well as data on our categorizations, are available in Supplementary file 2.

## Results

**Fig. 1:**
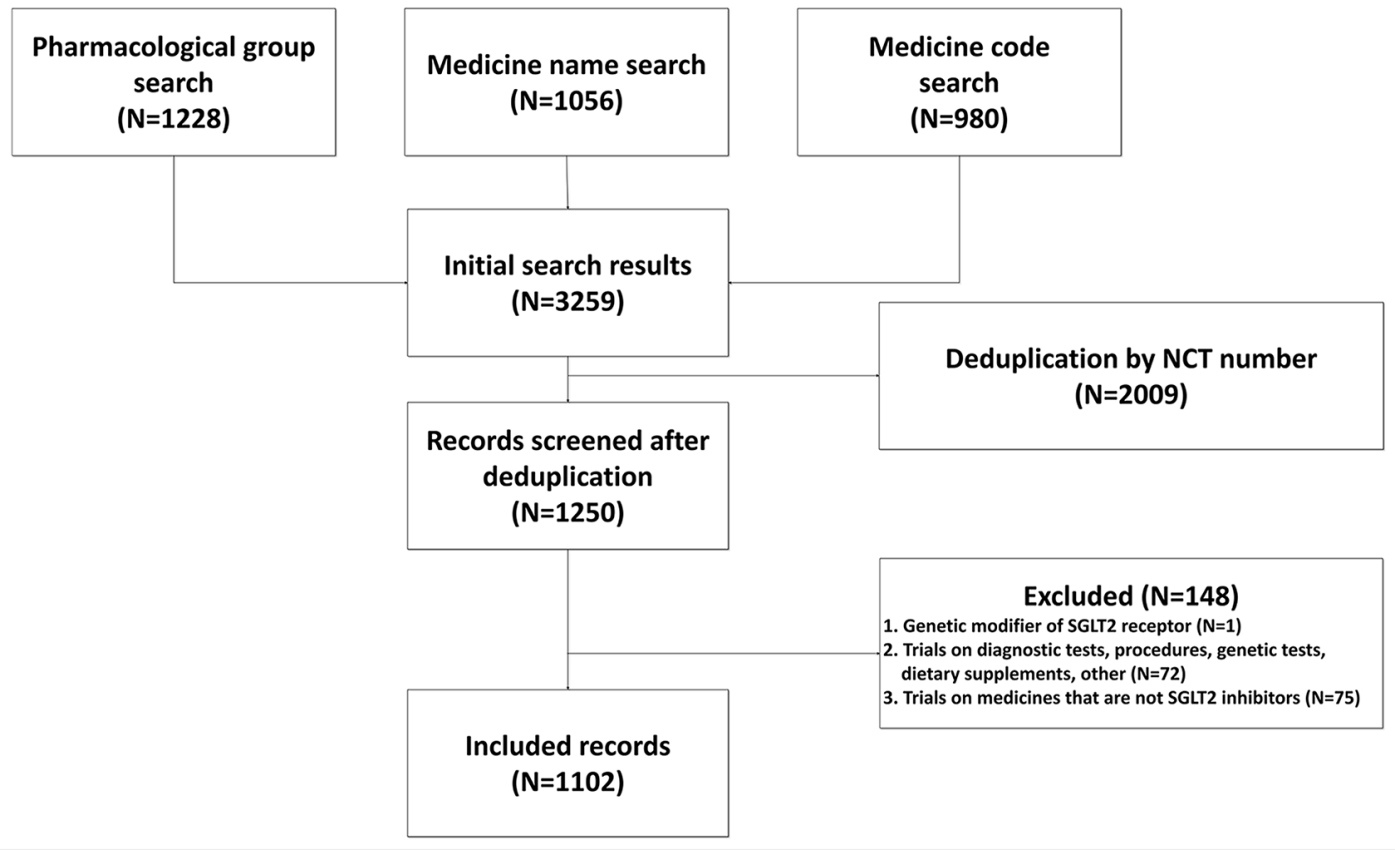
Flow diagram of study

The search of ClinicalTrials.gov retrieved 3259 registered trials. After deduplication and applying exclusion criteria, we excluded 148 trials (Supplementary Table 2). Reasons for excluding individual records are shown in Supplementary Table 3. We included 1102 trials in the final analysis. The flow diagram of records’ search, screening, and inclusion is shown in Fig. 1.

### Timeline of registrations

The first two interventional trials on SGLT2 inhibitors were registered in 2005, and the first observational trial in 2013. Since then, the number of registrations has progressed continuously, with several peaks. In 2022, up to the beginning of November, there were 128 registered trials on SGLT2 inhibitors (Supplementary Fig. 1).

The majority of trials complied with FDAAA 801 mandated registration deadlines (*N* = 752, 68%). FDAAA 801 compliant trials were registered a month before the trial start (median = 29.5 days, IQR = 0–96 days). Trials that failed to comply with FDAAA 801 deadline were registered more than 3 months after the beginning of the trial (median = 106.5 days, IQR = 39.25–356.5 days). Several trials started before the FDAAA 801 in 2007, so these trials were excluded from this sub-analysis.

### Trial characteristics

Most registered trials were interventional (*N* = 993, 90.1%), and more than 61% were completed (*N* = 676). Most trials were in Phase 1 (*N* = 287; 28.9%), Phase 3 (*N* = 233; 23.5%) or Phase 4 (*N* = 243; 24.5%). Almost all trials included both sexes, 53 (4.8%) trials included only men and 16 (1.5%) trials only women. The included population in most trials were adults, and 43 trials (3.9%) included children (< 18 years) (Table 2).

**Table 2:** Characteristics of clinical trials on SGLT2 inhibitors registered on ClinicalTrials.gov

Characteristics					*N*		%
Study type	Interventional				993		90.1
	Observational				109		9.9
Status	Completed				676		61.3
	Recruiting				159		14.4
	Unknown status				86		7.8
	Not yet recruiting				78		7.1
	Active, not recruiting				38		3.4
	Terminated				31		2.8
	Withdrawn				23		2.1
	Enrolling by invitation				10		0.9
	Suspended				1		0.1
Phase	Phase 1				287		28.9
	Phase 4				243		24.5
	Phase 3				233		23.5
	Phase 2				139		14
	Not applicable				46		4.6
	Phase 2/3				27		2.7
	Early phase 1				9		0.9
	Phase 1/2				9		0.9
Gender	All sexes				1033		93.7
	Male				53		4.8
	Female				16		1.5
Age	Adult + older adult				804		73
	Adult				248		22.5
	Child + adult + older adult				24		2.2
	Child + adult				12		1.1
	Child				7		0.6
	Older adult				7		0.6
Documents available on ClinicalTrials.gov	Not available				960		87.1
	Study protocol + statistical analysis				120		10.9
	Study protocol + statistical analysis plan + informed consent form				12		1.1
	Study protocol				4		0.4
	Informed consent form				3		0.3
	Statistical analysis plan				2		0.2
	Study protocol + informed consent form				1		0.1
Overall availability of results	Total	No results available		Has results			
		N	%	N		%	
Completed	676	399	59	277		41	
Terminated	31	21	67.7	10		32.3	
Withdrawn	23	23	100	0		0	
Unknown status	81	81	100	0		0	
Compliance with the mandatory posting of results (FDAAA 801)							
Completed	112	5	6.4	107		95.5	
Terminated	5	2	40	3		60	
Withdrawn	5	5	100	0		0	
Unknown status	2	2	100	0		0	
Total	124	14	11.3	110		88.7	

There were 23 withdrawn trials, with no enrolled participants; 4 did not provide the reason for withdrawal, 12 were withdrawn for the lack of funding or by sponsors’ decision, 2 trials were not granted approval by regulatory bodies, and 3 were withdrawn for technical issues (COVID-19 pandemic, incorrect trial registration). Out of 31 terminated trials, 14 were terminated due to recruitment challenges, 9 due to sponsors’ decision, and 4 due to COVID-19 pandemic.

### Funding

More than half of all interventional trials (*N* = 529; 53.3%), and observational trials (*N* = 55; 50.5%) were funded solely by industry, and another 12% (*N* = 120) of interventional trials and 8% (*N* = 9) of observational trials are funded partially by industry (in collaboration with other institutes). In total, almost 65% of all clinical trials were industry-funded.

### Availability of results and documents

Significant differences were found between results availability based on funding (Supplementary Table 4). Trials partially or fully funded by the industry posted results more frequently compared to the trials without industry funding (46.1% vs. 11.2%; *p* < 0.001) (Supplementary Tables 4 and 5).

Among 811 trials that reached “Primary completion date,” only 287 (35%) posted the results on ClinicalTrials.gov. After identifying applicable trials subject to FDAAA 801 regulation (*N* = 124), 110 (88%) complied with a legal obligation to post the results (Table 2.). In comparison, 177 trials (22%) published the results voluntarily, without legal obligation (χ = 281, *p* < 0.001).

Supplementary documents (e.g., study protocol, statistical analysis plan, informed consent) were unavailable for 87.1% (*N* = 960) of trials (Table 2). Since the implementation of FDAAA 801 (CFR 42 Part 11) in 2017, there were only 64 trials with the obligation to publish the supplementary documents, and 51 (80%) of them complied. However, 91 (9%) trial submitted the documents voluntarily. After the implementation of CFR 42, there has been an increase in the availability of documents, especially in voluntary submissions (4 trials vs. 87 trials).

### Trial design

Among observational trials, the majority were cohort studies (*N* = 81; 74.3%) (Table 3). Most interventional trials were randomized (*N* = 841; 84.7%), designed as parallel group (*N* = 626; 63%) or crossover (*N* = 222; 22.4%). No masking was performed in 444 trials (44.7%). Trials that used masking were most commonly double (*N* = 215; 21.7%) or quadruple masked (*N* = 196, 19.7%). The primary purpose of most interventional trials was treatment (*N* = 805; 81.1%) (Table 3). Median duration (Start date to Primary completion date) of observational trials was 20 months (IQR = 12–37), and 18 months (IQR = 7–28) for interventional trials.

**Table 3:** Study design of clinical trials on SGLT2 inhibitors registered on ClinicalTrials.gov

Observational		N	%
		109	9.9
Observational Model	Cohort	81	74.3
	Case-control	17	15.6
	Other	9	8.3
	Case only	2	1.8
Time perspective	Prospective	55	50.5
	Retrospective	48	44.0
	Other	4	3.7
	Cross-sectional	2	1.8
**Interventional**		No	%
		993	90.1
Allocation	Randomized	841	84.7
	N/A	104	10.5
	Non-randomized	48	4.8
Intervention model	Parallel assignment	626	63
	Crossover assignment	222	22.4
	Single group assignment	132	13.3
	Sequential assignment	8	0.8
	Factorial assignment	5	0.5
Masking	None	444	44.7
	Double	215	21.7
	Quadruple	196	19.7
	Triple	103	10.4
	Single	35	3.5
Masking by subgroups	No masking	444	44.7
	Participant + Care provider + Investigator + Outcome assessor	196	19.7
	Participant + Investigator	192	19.3
	Participant + Care provider + Investigator	77	7.8
	Participant + Investigator + Outcome assessor	21	2.1
	Participant	20	2
	Participant + Care provider	13	1.3
	Outcome assessor	8	0.8
	Participant + Outcome Assessor	7	0.7
	Investigator	6	0.6
	Participant + Care provider + Outcome assessor	5	0.5
	Investigator + Outcome Assessor	3	0.3
Primary purpose	Treatment	805	81.1
	Basic science	72	7.3
	Prevention	39	3.9
	Not Defined	34	3.4
	Other	31	3.1
	Diagnostic	6	0.6
	Health service research	4	0.4
	Screening	2	0.2

### Medical indications

**Fig. 2:**
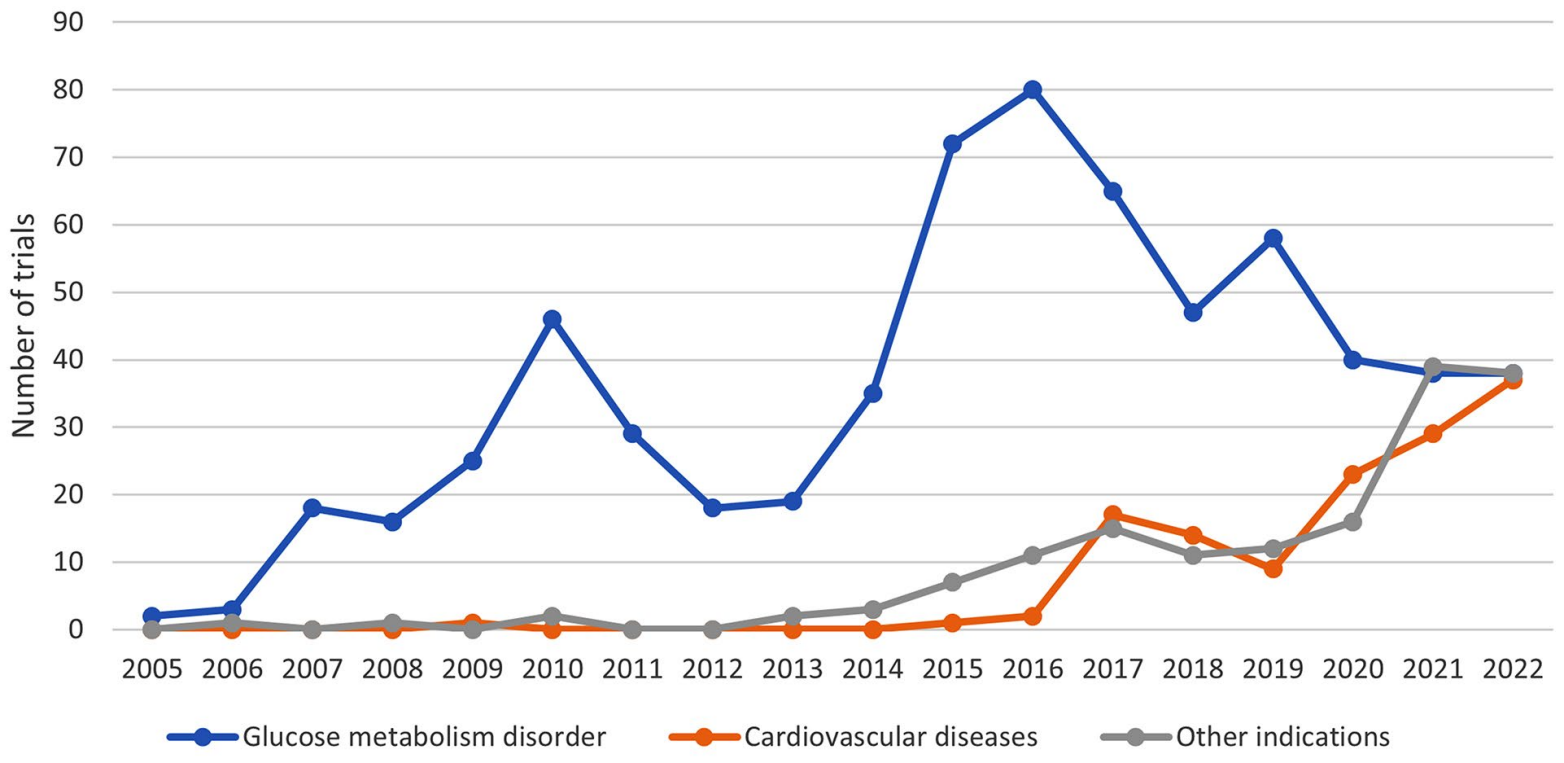
Evolution of indications for SGLT2 inhibitors by group on ClinicalTrials.gov

The majority of trials studied one (*N* = 839; 76.1%) medical condition, (Table 4). There were 145 trials performed in healthy subjects (13.2%). More than 60% of the trials included diabetic participants. Participants with heart diseases were studied in 12% of all trials (HF – *N* = 83; 7.5%) (Table 4). From 2005 up to 2015, trials in diabetes have dominated in numbers. After 2015, the number of trials in cardiovascular and renal disorders increased, and matched the number of trials in diabetes in 2022 (Fig. 2) (Supplementary Fig. 2).

**Table 4:** Summary of conditions studied in clinical trials on SGLT2 inhibitors registered on ClinicalTrials.gov

Conditions studied		No	%
Number of conditions studied	0	2	0.2
	1	839	76.1
	2	197	17.9
	3	43	3.9
	4	14	1.3
	5	5	0.5
	6	1	0.1
	7	1	0.1
Primary studied condition	Diabetes Mellitus Type 2	586	53.2
	Healthy	145	13.2
	Heart Failure	83	7.5
	Kidney diseases	61	5.5
	Heart diseases other	47	4.3
	Diabetes Mellitus Type 1	45	4.1
	Diabetic complications, Glucose metabolism disorder	34	3.1
	Liver Diseases	28	2.5
	Other	24	2.2
	Obesity	18	1.6
	Metabolical diseases	12	1.1
	Endocrinological diseases	9	0.8
	Cancer/Oncology	7	0.6
	Cardiomyopathy	3	0.3
	Total	1102	100
Secondary studied condition	Diabetes Mellitus Type 2	46	17.6
	Heart diseases other	45	17.2
	Kidney diseases	39	14.9
	Heart Failure	31	11.9
	Diabetic complications, Glucose metabolism disorder	30	11.5
	Other	19	7.3
	Liver Diseases	16	6.1
	Obesity	11	4.2
	Healthy	9	3.4
	Cancer/Oncology	6	2.3
	Metabolical diseases	6	2.3
	Cardiomyopathy	2	0.8
	Endocrinological diseases	1	0.4
	Total	261	100

### SGLT2 inhibitors and comparators

The most commonly studied medicines were dapagliflozin (*N* = 383; 34.8%), empagliflozin (*N* = 316; 28.7%) and canagliflozin (*N* = 127; 11,5%), accounting for 75% of all trials. Ipragliflozin, sotagliflozin and ertugliflozin were studied in 13% of trials (4.8%, 4.4%, and 3.1%). In 20 (1.9%) trials, specific SGLT2 inhibitors were not defined – the trial registrations simply wrote “SGLT2 inhibitors”. In 29 (2.5%) trials, multiple, distinct SGLT2 inhibitors were studied (Supplementary Table 6).

Most observational trials on SGLT2 inhibitors used no comparators (*N* = 62; 56.9%), and 30 trials used other hypoglycemic medications (27.5%). In interventional trials, placebo was the most frequent comparator (*N* = 299; 30.1%), while hypoglycemic medications were used in 142 trials (14.3%) (Supplementary Table 7). In total, 171 (17.2%) of interventional trials that used no comparator, 49% were in Phase 1 or Phase 2, and 38% were in Phase 3 or Phase 4.

### Sample size of trials

Sample size varied depending on the trial type. In 109 included observational trials, there were more than 9.5 million participants (median = 1,424; IQR = 367–27,897). Completed observational trials included 5.5 million participants (median = 3,061.5; IQR = 465.25–29,139.5). Prospective observational trials (*N* = 55) included 446,503 participants, while retrospective trials analyzed more than 9 million participants.

In 972 interventional trials, there were 312,124 participants (median = 63; IQR = 30–196.5). There were many smaller trials (95th percentile = 1182.1) and a few large trials with several thousand participants. The largest interventional trials on SGLT2 inhibitors are displayed in Supplementary Table 8.

## Discussion

We analyzed 1102 trials on 14 different SGLT2 inhibitors registered from 2005 to November 2022 on ClinicalTrials.gov. The first study was registered in September 2005, and since then the number of registrations increased progressively with several peaks. We confirmed our initial hypothesis that more than 50% of trials analyzed empagliflozin and dapagliflozin, were interventional trials, studied diabetes mellitus, were industry funded. Only one hypothesis can be partially rejected, regarding study results availability; even though only 35% of trials posted the results, in a group of trials subject to the FDAAA 801 regulation, 88% posted the results publicly.

The majority of registered trials were interventional. However, despite being fewer in numbers, observational trials included more than 9 million participants, compared to 300,000 in interventional trials. Most trials were completed (62%); about 25% were in the early stages (recruitment has not started yet or recruiting). Trials in diabetes accounted for more than 60% of all trials, followed by trials in heart diseases (12%). Dapagliflozin, empagliflozin and canagliflozin were studied in 75% of trials.

In our study, almost 65% of all trials were industry-funded, and those trials were more likely to have their results publicly available. This finding is in line with previously published research by Zwierzyna et al., which analyzed 45 620 clinical trials completed by July 2015 [23]. While this is welcoming improvement, more regulatory and independent oversight is needed [24], as industry funded research is more likely to have biased design (e.g. inadequate comparators) or interpretation (favorable spin), ghost authorship and industry-based statistical analysis [25]. Finally, although we have shown how funding can impact the availability of results, care is needed when interpreting these findings due to changes in rules for mandatory reporting.

In 2012, Prayle et al. published their analysis of compliance with mandatory reporting of clinical trial results on ClinicalTrials.gov. Of these, only 22% reported the results as mandated, and 10% of trials reported results voluntarily [26]. In a similar study published in 2020, 40% of applicable trials submitted the results within a 1-year deadline, and 64% at any time [27]. In our study, 88% of trials subject to mandatory reporting have posted the results, compared to 18% of trials which voluntarily posted the results. Mandatory reporting is regulated by the CFR 42, which warrants registration and results reporting of clinical trials in the Clinicaltrials.gov database. This regulation aims to enhance transparency and accountability by ensuring that trial information is publicly accessible and that results are disclosed in a timely manner. The implementation of CFR 42 impacts various aspects of clinical trial reporting, including the completeness and timeliness of data submissions, which can introduce potential biases and affect the overall integrity of clinical trial databases [16]. Consequently, compliance with CFR 42 is a significant factor to consider when analyzing study characteristics and interpreting results within the context of clinical trials.

Results of our study show significant improvement in mandatory result posting. Reporting of results for trials without legal obligation remains low but is improving. We also observed an increasing number of voluntary submissions of supplementary documents. While any step towards increased transparency is beneficial, this signals that we still have to rely on regulatory measures to reliably obtain transparency and opens the question of potential benefits of extending the criteria for mandatory reporting of results. Large observational trials offer external validity and often study patients under real-world conditions, which is valuable for clinical practice guidelines and directly influences decision making, further highlighting the importance of this question [28].

Based on the current numbers from ClinicalTrials.gov (April 2023), there are more than 445,000 registered trials on ClinicalTrials.gov, and observational studies account for 22% [29]. For comparison, in our study, 10% of trials were labeled as observational. This discrepancy suggests a strong registration bias.

While the first natural-occurring compound to induce glucosuria was phlorizin, isolated in 1835, it was not suitable for clinical use as it is non-selective, has a low oral bioavailability and can cause serious side effects [30]. First developed SGLT2 inhibitor was dapagliflozin [31], followed by empagliflozin and canagliflozin. These drugs were initially used for type 2 DM, and this remains the primary medical condition in Clinicaltrials.gov today. In 2009, FDA recommended conducting long-term cardiovascular outcome trials (CVOT) to examine the safety of new anti-diabetic medicines [32–34]. Three large CVOT trials were conducted on SGLT2 inhibitors: EMPA-REG on empagliflozin (2010), DECLARE–TIMI 58 on dapagliflozin (2012) and CANVAS-R on canagliflozin (2013) [5, 6, 35]. Positive results of these trials prompted further research. After 2013, we witness an increasing number of trials in heart and kidney diseases, and not just in SGLT2 inhibitors. For example, glucagon-like peptide-1 (GLP-1) receptor agonists are a class of medications developed for treatment of type 2 DM [36]. While initially used in treatment of DM2, their trajectory is similar to SGLT2 inhibitors. Major CVOTs have shown how GLP-1 receptor agonists reduce the risk of major adverse cardiovascular events and are now used in the treatment of obesity, and they show a number of positive biological effects [36]. Studies are underway to explore the effects of GLP-1 receptor agonists in sleep apnea, Alzheimer’s disease, kidney disease and substance-use disorder [37]. We expect more cardiovascular breakthroughs from drugs developed in other fields in the future, and robust and transparent reporting of outcomes is necessary to facilitate this process.

SGLT2 inhibitors are recommended in clinical practice guidelines for patients with HF with reduced ejection fraction [38] (class of recommendation I, level of evidence A). European Society of Cardiology defined main outcomes in HF treatment, which include mortality (cardiovascular and all-cause), hospitalizations, improvement in clinical status, functional capacity, and quality of life [39]. Current evidence supports their use in HF with preserved ejection fraction, but with modest benefits only for the outcome of hospitalization [40]. To support further use of SGLT2 inhibitors in cardiovascular diseases, more trials that evaluate these outcomes are necessary.

### Limitations

We analyzed only one trial registry (ClinicalTrials.gov), considering it is the world’s largest and most complete clinical trial registry. Medicines were identified prior to creating our search strategy. However, two additional SGLT2 inhibitors were identified during the coding of our database, so the initial search protocol was amended to incorporate them. Despite the extensive search strategy, there is a possibility that some registrations were not included.

Additionally, registration of observational trials is not mandatory by FDA, and there are likely many observational trials on SGLT2 inhibitors that were not registered in ClinicalTrials.gov and could not be analyzed. Even though ClinicalTrials.gov implemented registration of observational trials since its availability to the public (February 2000) and modified it for the input of specific data for observational trials (October 2007), the number of registered observational trials remained low [41]. Considering that observational trials included in our research involved more than 9 million participants, it is reasonable to expect there is a substantial number of participants in non-registered observational trials. While this might be important for overall effect size estimates, in our study we focused on the design of trials that were required to register for approval of use.

Registration of trials was not mandatory by the FDAAA 801 prior to September 27, 2007, which could lead to misinterpretation of trial timelines as well as the exclusion of trials conducted prior to September 2007 and particularly influence industry sponsored trials. Incorrectly exported data from ClinicalTrials.gov were manually corrected; however, there is a possibility that some mistakes were not recognized. For transparency, we published all raw data collected within this study and categorizations to enable verification.

### Generalizability

This trial represents, to our knowledge, the only comprehensive study of registered trials on SGLT2 inhibitors, as no similar studies have been found in published literature. By maximizing the inclusion criteria, we aimed to gain thorough insight into registration landscape of this newly developed medicine class. This study offers a synoptic view of developed medications, methodology, and characteristics of trials on SGLT2 inhibitors. The change of medical conditions in which SGLT2 inhibitors were researched through the years indicates the importance of performing large CVOTs, not only to prevent serious adverse CV events but to identify potential new applications of existent medications.

## Conclusion

The first clinical trial on an SGLT2 inhibitor was registered in 2005, and since then, 14 different medications have emerged. The number of trials is increasing progressively with expanding the indications for its use, shifting from DM to cardiovascular and renal diseases. Interventional design was used in most trials; however, observational trials are increasing in number as well as participant enrolment. Influence of observational research warrants further investigation, with potential registration regulatory changes. The majority of trials were sponsored by the pharmaceutical industry, and reporting of results shows modest improvement.

### Electronic supplementary material

Below is the link to the electronic supplementary material.

Supplementary Material 1

Supplementary Material 2

Supplementary Material 3

Supplementary Material 4

## Data Availability

The datasets supporting the conclusions of this article are included within the article and its additional files. Supplementary tables are attached in Supplementary file 1. All raw data collected within this study are included in Supplementary file 2.
