# Supplementary figures and images for: Methodology of clinical trials on sodium-glucose cotransporter 2 inhibitors registered on ClinicalTrials.gov: a cross-sectional study
Source: BMC Med Res Methodol. 2024 Jul 30;24:164. doi: 10.1186/s12874-024-02292-5 (PMC11289909; doi:10.1186/s12874-024-02292-5)

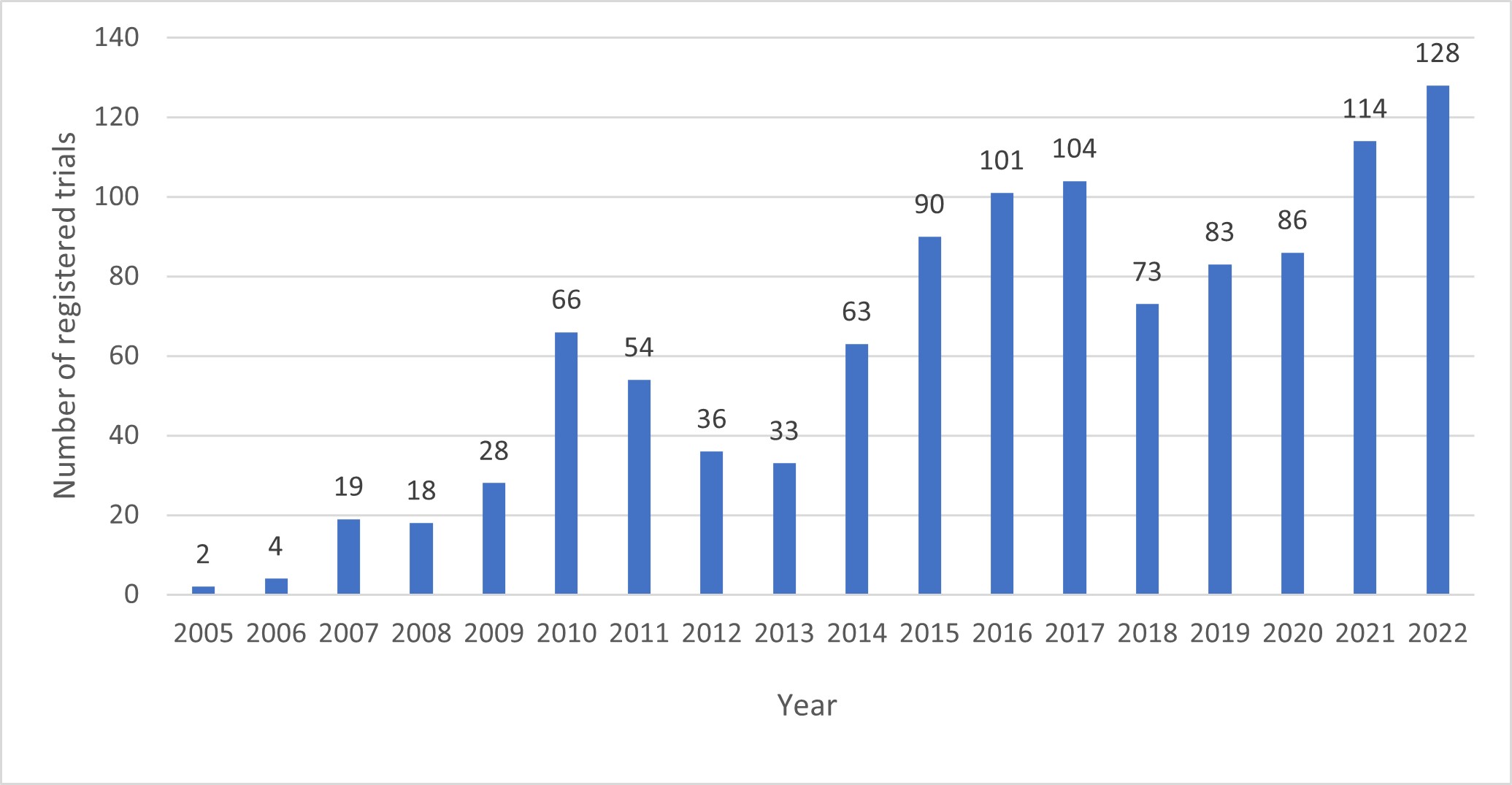

Supplement: Supplementary file 1 — Supplementary Material 1 [file 12874_2024_2292_MOESM1_ESM.jpg]

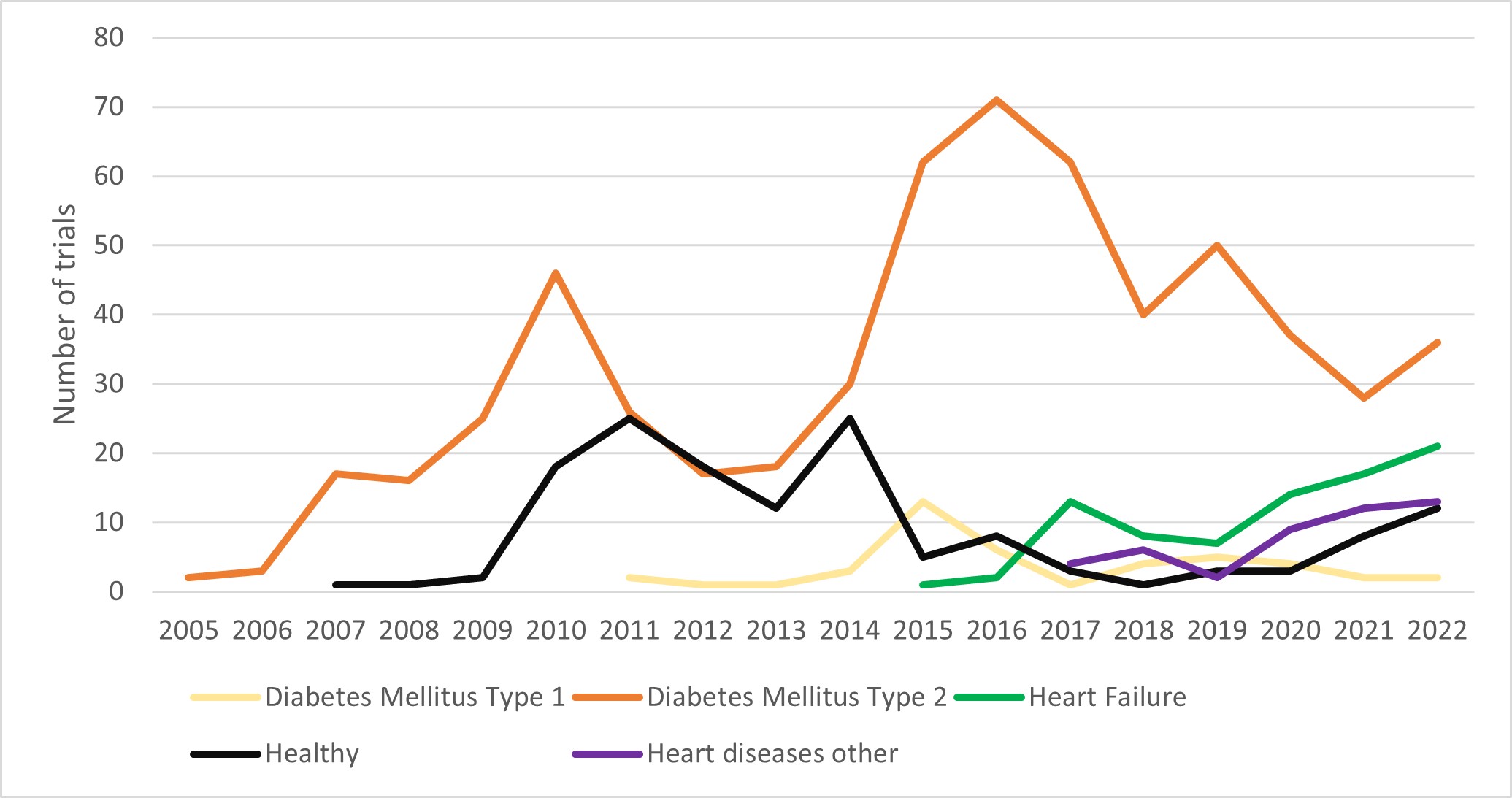

Supplement: Supplementary file 2 — Supplementary Material 2 [file 12874_2024_2292_MOESM2_ESM.jpg]
