# Supplementary material for: Methodology of clinical trials on sodium-glucose cotransporter 2 inhibitors registered on ClinicalTrials.gov: a cross-sectional study
Source: BMC Med Res Methodol. 2024 Jul 30;24:164. doi: 10.1186/s12874-024-02292-5 (PMC11289909; doi:10.1186/s12874-024-02292-5)
Supplement: Supplementary file 3 — Supplementary Material 3 [file 12874_2024_2292_MOESM3_ESM.docx]

**Supplementary File 1**

Contents

[Supplementary Table 1. Search strategy used in the study 2](#_Toc135346383)

[Supplementary Table 2. Summarized reasons for excluding records 3](#_Toc135346384)

[Supplementary Table 3. Reasons for excluding individual records 4](#_Toc135346385)

[Supplementary Table 4. Availability of trial results on ClinicalTrials.gov based on funding 6](#_Toc135346386)

[Supplementary Table 5. Differences between the availability of trial results on ClinicalTrials.gov based on funding 7](#_Toc135346387)

[Supplementary Table 6. Frequency of SGLT2 inhibitors studied in registered trials on ClinicalTrials.gov 8](#_Toc135346388)

[Supplementary Table 7. Summary of comparators used in observational and interventional trials 9](#_Toc135346389)

[Supplementary Table 8. The list of largest interventional trials on SGLT2 inhibitors registered on ClinicalTrials.gov 10](#_Toc135346390)

[Supplementary Figure 1. Number of registered trials on SGLT2 inhibitors by year on ClinicalTrials.gov 11](#_Toc135346391)

[Supplementary Figure 2. Evolution of indications for SGLT2 inhibitors registered on ClinicalTrials.gov 11](#_Toc135346392)

# Supplementary Table 1. Search strategy used in the study

| Search arm | PHARMACOLOGICAL GROUP | GENERIC NAME | MEDICINE CODE NAME |
| --- | --- | --- | --- |
| Keywords | **SGLT2** OR **SGLT2 inhibitors** OR **sodium glucose transporter 2** OR **sodium glucose cotransporter 2** | **dapagliflozin** OR **empagliflozin** OR **canagliflozin** OR **ipragliflozin** OR **tofogliflozin** OR **luseogliflozin** OR **ertugliflozin** OR **bexagliflozin** OR **remogliflozin** OR **licogliflozin** OR **sotagliflozin** OR **sergliflozin** OR **enavogliflozin** OR **henagliflozin** | **BMS-512148** OR **BI 10773** OR **JNJ-28431754** OR **TA-7284** OR **ASP1941** OR **CSG452** OR **TS-071** OR **898537-18-3** OR **PF-04971729** OR **MK-8835** OR **EGT0001442** OR **EGT0001474** OR **GSK189075** OR **LIK066** OR **LX4211** OR **GW869682** OR **DWP16001** OR **DWP16001** OR **DWP-16001** OR **SHR-3824** |
| Results by group | N=1228 | N=1056 | N=980 |
| Initial search results | N=3234 | | |
| Included records | N=1102 | | |

# Supplementary Table 2. Summarized reasons for excluding records

| **Reasons for exclusion** | **N** | **%** |
| --- | --- | --- |
| Other anti-diabetic medications | 60 | 40.5 |
| Other* | 26 | 17.6 |
| Diagnostic test | 23 | 15.5 |
| Behavioural test | 11 | 7.4 |
| Other medications | 10 | 6.8 |
| Devices | 8 | 5.4 |
| Procedure | 7 | 4.7 |
| Genetic test | 2 | 1.4 |
| Genetic modifier of SGLT2 receptor | 1 | 0.7 |
| Total | 148 | 100 |

*Category “Other” includes dietary supplements, educational intervention, prescription protocol, etc. For details, please see raw data in Supplementary file 2.

# Supplementary Table 3. Reasons for excluding individual records

| **N** | **NCT Number** | **Reasons for exclusion** | **N** | **NCT Number** | **Reasons for exclusion** |
| --- | --- | --- | --- | --- | --- |
| 1 | NCT05612269 | diagnostic test | 79 | NCT05495516 | Genetic |
| 2 | NCT05520684 | diagnostic test | 80 | NCT04621656 | devices |
| 3 | NCT05341388 | diagnostic test | 81 | NCT02730377 | other anti-diabetic medications |
| 4 | NCT05164523 | diagnostic test | 82 | NCT05040087 | other |
| 5 | NCT05402579 | genetic test | 83 | NCT05433220 | other |
| 6 | NCT05417646 | diagnostic test | 84 | NCT03882970 | other anti-diabetic medications |
| 7 | NCT02964247 | other anti-diabetic medications | 85 | NCT05021419 | other |
| 8 | NCT03717207 | diagnostic test | 86 | NCT05426018 | other anti-diabetic medications |
| 9 | NCT02597049 | other anti-diabetic medications | 87 | NCT05198284 | diagnostic test |
| 10 | NCT03560323 | other anti-diabetic medications | 88 | NCT04960514 | diagnostic test |
| 11 | NCT03557138 | diagnostic test | 89 | NCT04791358 | diagnostic test |
| 12 | NCT04862858 | other | 90 | NCT05180773 | other medications |
| 13 | NCT03965000 | other anti-diabetic medications | 91 | NCT04237701 | devices |
| 14 | NCT02773368 | other anti-diabetic medications | 92 | NCT05413447 | devices |
| 15 | NCT04791826 | behavioural test | 93 | NCT04445181 | other |
| 16 | NCT02787551 | other, SGLT2 only as comparator | 94 | NCT03430284 | behavioural test |
| 17 | NCT05606913 | other anti-diabetic medications | 95 | NCT04061200 | other anti-diabetic medications |
| 18 | NCT03188887 | other, SGLT2 only as comparator | 96 | NCT04170543 | other anti-diabetic medications |
| 19 | NCT00836225 | Genetic modifier of SGLT2 receptor | 97 | NCT03560375 | behavioural test |
| 20 | NCT03878459 | other anti-diabetic medications | 98 | NCT04885712 | other anti-diabetic medications |
| 21 | NCT05526157 | other anti-diabetic medications | 99 | NCT05226897 | other anti-diabetic medications |
| 22 | NCT03086330 | other anti-diabetic medications | 100 | NCT04170998 | other anti-diabetic medications |
| 23 | NCT02105792 | other anti-diabetic medications | 101 | NCT04492722 | other anti-diabetic medications |
| 24 | NCT03951805 | other anti-diabetic medications | 102 | NCT05028140 | other anti-diabetic medications |
| 25 | NCT05463705 | behavioural test | 103 | NCT05342545 | other |
| 26 | NCT05531214 | behavioural test | 104 | NCT03861039 | other anti-diabetic medications |
| 27 | NCT03922750 | other anti-diabetic medications | 105 | NCT05578352 | other anti-diabetic medications |
| 28 | NCT05330221 | behavioural test | 106 | NCT04416269 | other anti-diabetic medications |
| 29 | NCT05413369 | other anti-diabetic medications | 107 | NCT04809220 | other anti-diabetic medications |
| 30 | NCT04872959 | diagnostic test | 108 | NCT02682680 | other medications |
| 31 | NCT05535322 | other anti-diabetic medications | 109 | NCT05161793 | devices |
| 32 | NCT03007329 | other anti-diabetic medications | 110 | NCT05037058 | other |
| 33 | NCT02041234 | procedure | 111 | NCT05564572 | diagnostic test |
| 34 | NCT05001165 | other | 112 | NCT04735354 | other medications |
| 35 | NCT04272359 | other anti-diabetic medications | 113 | NCT02844153 | other |
| 36 | NCT04768322 | devices | 114 | NCT03736668 | diagnostic test |
| 37 | NCT04572165 | other anti-diabetic medications | 115 | NCT03365063 | other |
| 38 | NCT05181631 | other | 116 | NCT05419583 | diagnostic test |
| 39 | NCT05295368 | other | 117 | NCT04162067 | other |
| 40 | NCT04450394 | other anti-diabetic medications | 118 | NCT02954692 | other anti-diabetic medications |
| 41 | NCT03730662 | other anti-diabetic medications | 119 | NCT04880005 | behavioural test |
| 42 | NCT02561078 | other anti-diabetic medications | 120 | NCT03766750 | other anti-diabetic medications |
| 43 | NCT05558904 | procedure | 121 | NCT04670666 | other medications |
| 44 | NCT05122741 | other medications | 122 | NCT01619059 | other anti-diabetic medications |
| 45 | NCT03932721 | other medications | 123 | NCT02577016 | other anti-diabetic medications |
| 46 | NCT03779217 | procedure | 124 | NCT04847531 | other |
| 47 | NCT03804411 | other | 125 | NCT04603508 | other medications |
| 48 | NCT04146155 | other anti-diabetic medications | 126 | NCT03909555 | other |
| 49 | NCT04446026 | other anti-diabetic medications | 127 | NCT04602754 | other medications |
| 50 | NCT05182359 | diagnostic test | 128 | NCT04182464 | other medications |
| 51 | NCT05275608 | behavioural test | 129 | NCT03566693 | devices |
| 52 | NCT03798054 | other anti-diabetic medications | 130 | NCT03588000 | Devices |
| 53 | NCT04626323 | procedure | 131 | NCT04903717 | behavioural test |
| 54 | NCT03678727 | exercise test | 132 | NCT04559191 | devices |
| 55 | NCT04514458 | diagnostic test | 133 | NCT03444584 | other anti-diabetic medications |
| 56 | NCT04931030 | behavioural test | 134 | NCT05405556 | diagnostic test |
| 57 | NCT04128995 | procedure | 135 | NCT02304081 | other anti-diabetic medications |
| 58 | NCT05035459 | diagnostic test | 136 | NCT03842267 | other anti-diabetic medications |
| 59 | NCT04573478 | other medications | 137 | NCT02053116 | other anti-diabetic medications |
| 60 | NCT05268081 | educational | 138 | NCT05296044 | other anti-diabetic medications |
| 61 | NCT04573764 | Dietary supplement | 139 | NCT05101135 | other anti-diabetic medications |
| 62 | NCT02109978 | other | 140 | NCT05144360 | other anti-diabetic medications |
| 63 | NCT02221284 | other anti-diabetic medications | 141 | NCT04027023 | other anti-diabetic medications |
| 64 | NCT05284344 | other anti-diabetic medications | 142 | NCT05504239 | other anti-diabetic medications |
| 65 | NCT05349955 | other | 143 | NCT05504226 | other anti-diabetic medications |
| 66 | NCT04802395 | diagnostic test | 144 | NCT05017987 | other anti-diabetic medications |
| 67 | NCT05572957 | procedure | 145 | NCT03849261 | other anti-diabetic medications |
| 68 | NCT04633005 | other | 146 | NCT03646799 | other anti-diabetic medications |
| 69 | NCT05147818 | diagnostic test | 147 | NCT05380596 | behavioural test |
| 70 | NCT04686604 | other | 148 | NCT04063098 | diagnostic test |
| 71 | NCT03214380 | other anti-diabetic medications |  |  |  |
| 72 | NCT04841096 | other anti-diabetic medications |  |  |  |
| 73 | NCT03832595 | other |  |  |  |
| 74 | NCT05132244 | procedure |  |  |  |
| 75 | NCT05583513 | diagnostic test |  |  |  |
| 76 | NCT04724837 | other anti-diabetic medications |  |  |  |
| 77 | NCT05284682 | diagnostic test |  |  |  |
| 78 | NCT04079881 | other anti-diabetic medications |  |  |  |

# Supplementary Table 4. Availability of trial results on ClinicalTrials.gov based on funding

| **Funded by** |  | **No results available** | **Has results** | **Total** |
| --- | --- | --- | --- | --- |
| Industry | Count | 278 | 232 | 510 |
|  | % within row | 54.5 % | 45.5 % | 100.0 % |
| NIH | Count | 1 | 1 | 2 |
|  | % within row | 50.0 % | 50.0 % | 100.0 % |
| Other | Count | 137 | 14 | 151 |
|  | % within row | 90.7 % | 9.3 % | 100.0 % |
| Industry + Other | Count | 44 | 34 | 78 |
|  | % within row | 56.4 % | 43.6 % | 100.0 % |
| NIH + Other | Count | 0 | 5 | 5 |
|  | % within row | 0.0 % | 100.0 % | 100.0 % |
| Industry + NIH + Other | Count | 1 | 0 | 1 |
|  | % within row | 100.0 % | 0.0 % | 100.0 % |
| Total | Count | 461 | 286 | 747 |
|  | % within row | 61.7 % | 38.3 % | 100.0 % |

# Supplementary Table 5. Differences between the availability of trial results on ClinicalTrials.gov based on funding

|  | **No results available** | **Has results** | **Total** | **χ^2^** | *P* |
| --- | --- | --- | --- | --- | --- |
| Industry | 278 | 232 | 516 | 55.87 | <0.001 |
|  | 54.5 % | 45.5 % | 100.0 % |  |  |
| Industry + other | 45 | 34 | 79 |  |  |
|  | 57.0% | 43.0% | 100 |  |  |
| NIH + Other | 138 | 20 | 158 |  |  |
|  | 87.3% | 12.7% | 100.00% |  |  |

# Supplementary Table 6. Frequency of SGLT2 inhibitors studied in registered trials on ClinicalTrials.gov

| **Study medicine** | No | % |
| --- | --- | --- |
| Dapagliflozin | 383 | 34.8 |
| Empagliflozin | 316 | 28.7 |
| Canagliflozin | 127 | 11.5 |
| Ipragliflozin | 53 | 4.8 |
| Sotagliflozin | 49 | 4.4 |
| Ertugliflozin | 34 | 3.1 |
| Any SGLT2 inhibitor | 20 | 1.8 |
| Bexagliflozin | 19 | 1.7 |
| Enavogliflozin | 18 | 1.6 |
| Empagliflozin/dapagliflozin/canagliflozin | 17 | 1.5 |
| Remogliflozin | 17 | 1.5 |
| Henagliflozin | 15 | 1.4 |
| Licogliflozin | 12 | 1.1 |
| Tofogliflozin | 6 | 0.5 |
| Empagliflozin/dapagliflozin | 5 | 0.5 |
| Empagliflozin/dapagliflozin/canagliflozin/ertugliflozin | 4 | 0.4 |
| YG1699 | 2 | 0.2 |
| Sergliflozin | 1 | 0.1 |
| Empagliflozin/dapagliflozin/canagliflozin/ ipragliflozin/tofogliflozin/luseogliflozin | 1 | 0.1 |
| Empagliflozin/dapagliflozin/canagliflozin/ licogliflozin/tofogliflozin/ipragliflozin | 1 | 0.1 |
| Luseogliflozin | 1 | 0.1 |
| Sergliflozin/remogliflozin | 1 | 0.1 |
| Total | 1102 | 100.0 |

# Supplementary Table 7. Summary of comparators used in observational and interventional trials

| **Observational** | **No** | **%** |
| --- | --- | --- |
|  | 109 | 9.9 |
| No comparator | 62 | 56.9 |
| Anti-diabetic therapy | 30 | 27.5 |
| SGLT2 + anti-diabetic therapy | 9 | 8.3 |
| SGLT2 | 4 | 3.7 |
| Anti-diabetic therapy + other | 1 | 0.9 |
| Other | 1 | 0.9 |
| SGLT2 + anti-diabetic therapy + other | 1 | 0.9 |
| SGLT2 + other | 1 | 0.9 |
| **Interventional** | **No** | **%** |
|  | 993 | 90.1 |
| Placebo | 299 | 30.1 |
| No comparator | 171 | 17.2 |
| Anti-diabetic therapy | 142 | 14.3 |
| Other | 86 | 8.7 |
| SGLT2 + anti-diabetic therapy | 84 | 8.5 |
| Placebo + anti-diabetic therapy | 62 | 6.2 |
| SGLT2 | 40 | 4 |
| SGLT2 + placebo | 40 | 4 |
| SGLT2 + placebo + anti-diabetic therapy | 21 | 2.1 |
| Placebo + other | 17 | 1.7 |
| SGLT2 + other | 15 | 1.5 |
| SGLT2 + placebo + other | 9 | 0.9 |
| Anti-diabetic therapy + other | 3 | 0.3 |
| SGLT2 + anti-diabetic therapy + other | 3 | 0.3 |
| Placebo + anti-diabetic therapy + other | 1 | 0.1 |

# Supplementary Table 8. The list of largest interventional trials on SGLT2 inhibitors registered on ClinicalTrials.gov

|  | **Trial name** | **Acronym** | **Enrolment** | **Study medication** |
| --- | --- | --- | --- | --- |
| 1 | Dapagliflozin and Cardiovascular Outcomes in Type 2 Diabetes | DECLARE-TIMI58 | 17190 | dapagliflozin |
| 2 | Sotagliflozin in Patients with Diabetes and Chronic Kidney Disease | SCORED | 10584 | sotagliflozin |
| 3 | Prevention of Cardiovascular and Diabetic kidney disease in Type 2 Diabetes | PRECIDENTD | 9000 | empagliflozin or dapagliflozin or canagliflozin |
| 4 | Cardiovascular Outcomes with Ertugliflozin in Type 2 Diabetes | VERTIS CV | 8246 | ertugliflozin |
| 5 | Empagliflozin, Cardiovascular Outcomes, and Mortality in Type 2 Diabetes | EMPA-REG OUTCOME | 7064 | empagliflozin |
| 6 | The Study of Heart and Kidney Protection with Empagliflozin | EMPA-KDINEY | 6609 | empagliflozin |
| 7 | Empagliflozin in patients post myocardial infarction rationale and design of the EMPACT-MI trial | EMPACT-MI | 6500 | empagliflozin |
| 8 | Dapagliflozin Effects on Cardiovascular Events in Patients with an Acute Heart Attack | DAPA-MI | 6400 | dapagliflozin |
| 9 | Dapagliflozin in Heart Failure with Mildly Reduced or Preserved Ejection Fraction | DELIVER | 6263 | dapagliflozin |
| 10 | Empagliflozin outcome trial in patients with chronic heart failure with preserved ejection fraction | EMPEROR-Preserved | 5988 | empagliflozin |
| 11 | Canagliflozin and Cardiovascular and Renal Events in Type 2 Diabetes | CANVAS-R | 5813 | canagliflozin |
| 12 | The Effect of Dapagliflozin on the Short-term Prognosis of Patients with Acute Myocardial Infarction |  | 5211 | dapagliflozin |
| 13 | Dapagliflozin in Patients with Heart Failure and Reduced Ejection Fraction | DAPA-HF | 4744 | dapagliflozin |
| 14 | Canagliflozin and Renal Outcomes in Type 2 Diabetes and Nephropathy | CREDENCE | 4401 | canagliflozin |
| 15 | Canagliflozin cardiovascular Assessment Study | CANVAS | 4330 | canagliflozin |
| 16 | Dapagliflozin in Patients with Chronic Kidney Disease | Dapa-CKD | 4304 | dapagliflozin |
| 17 | SGLT2 Inhibitor or Metformin as Standard Treatment of Early-Stage Type 2 Diabetes | SMARTEST | 4300 | dapagliflozin |
| 18 | Randomised Evaluation of COVID-19 Therapy | RECOVERY | 4150 | empagliflozin |
| 19 | Cardiovascular and Renal Outcomes with Empagliflozin in Heart Failure | EMPEROR-Reduced | 3730 | empagliflozin |
| 20 | Prevention With SGLT-2 Inhibition of Acute Kidney Injury in Intensive Care | PREVENTS-AKI | 3000 | dapagliflozin |

# Supplementary Figure 1. Number of registered trials on SGLT2 inhibitors by year on ClinicalTrials.gov

# Supplementary Figure 2. Evolution of indications for SGLT2 inhibitors registered on ClinicalTrials.gov
